# Multi‐Omics Comes of Age: Immune Profiling of Human Aging

**DOI:** 10.1111/imcb.70145

**Published:** 2026-07-03

**Authors:** Kirsten Skye Hicks, Connie Jackaman

**Affiliations:** ^1^ Curtin Medical Research Institute Curtin University Bentley Western Australia Australia; ^2^ School of Diagnostic and Therapeutic Sciences Curtin University Bentley Western Australia Australia

**Keywords:** aging, B cell, multi‐omics, T cell, vaccination

## Abstract

A recent study by Gong et al. maps peripheral immune aging using multi‐omic profiling and longitudinal influenza vaccination across adults aged 25–90+, revealing midlife (55–65) immune remodeling. Analyses of PBMCs, proteomics, and cell function show early dysregulation before aging, highlighting links to later disease susceptibility before inflammaging.

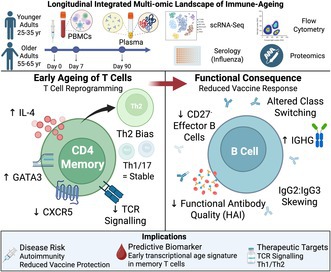

## 1 Introduction

**FIGURE 1:**
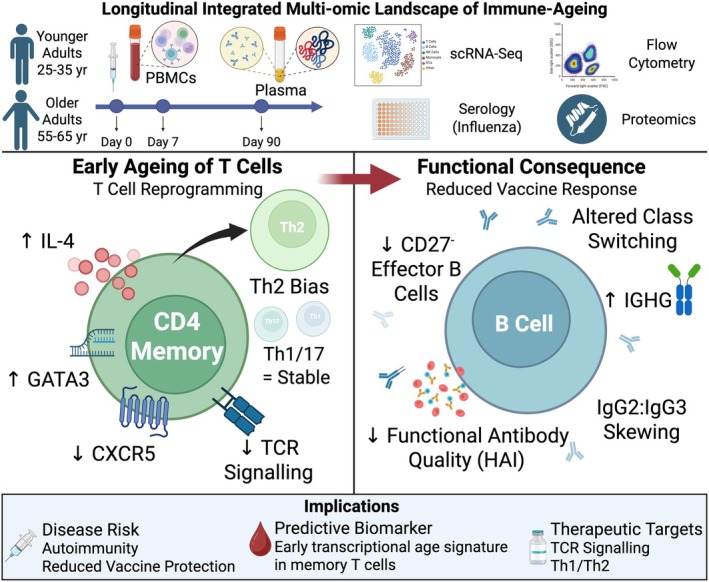
A recent study by Gong et al., maps peripheral immune aging using multi‐omic profiling and longitudinal influenza vaccination across adults aged 25–90+, revealing midlife (55–65) immune remodeling. Analyses of PBMCs, proteomics, and cell function show early dysregulation before aging, highlighting links to later disease susceptibility before inflammaging. Image generated in Biorender.

Advances in multi‐omics have recently been applied to aging research enabling integrated profiling of transcriptomics, epigenomics, proteomics, and immune repertoires at single‐cell resolution [1, 2]. A recent study has highlighted this approach by combining single‐cell RNA sequencing, proteomics, and functional assays to profile immune aging [1]. Through the use of advanced multi‐omic analysis, intrinsic aging processes can be decoupled from extrinsic influences such as inflammation and CMV, highlighting previously undiscovered signatures of immune remodeling (Figure 1).

## 2 Multi‐Omics Combined With Longitudinal Analysis of Immune Aging

This study provides a comprehensive view of peripheral immune aging combining longitudinal sampling post influenza vaccination with multi‐omic profiling across adults spanning pre‐, mid‐, and advanced aging [1]. Three age brackets were analyzed: younger adults (25–35 years), older adults (55–65 years) generally considered on the cusp of immune aging, and a follow‐up cohort aged ≥ 40 to 90+ years to validate aging signatures. Importantly, this study includes a midlife stage (55–65 years) which often coincides with a rising incidence of immune‐related conditions such as cardiovascular disease, neurodegenerative disorders, and autoimmunity. These observations suggest that gradual immune remodeling during this period may shape subsequent disease susceptibility and long‐term pathological trajectories as a recent concept separate to well‐characterized age‐related immune changes such as inflammaging [3]. This multi‐omic approach systematically describes how aging imprints on cellular, molecular and functional levels, providing evidence of immune dysregulation occurring prior to advanced aging (65+ years) and overt disease onset.

## 3 Decoupling From CMV, Immunosenescence and Inflammaging

Prior studies have suggested that CMV drives expansion of late or terminally differentiated T cells which could potentially promote a Th1 inflammatory bias [4]. Interestingly, in this study, controlling for CMV status uncovered an intrinsic Th2‐bias during aging, independent of CMV status. This study also showed that the age‐related shift in immune subsets was a result of the aging process and not necessarily a reflection of sex differences previously described in PBMCs from older individuals (age range young 22–40, middle 41–64, older 65+; [5]). Prior studies have suggested males have a faster shift toward inflammaging during aging leading to decreased T‐ and B‐cell responses, while these responses have a slower decline in females over time [5]. However, this study suggests that early age‐related T‐cell changes may not necessarily be driven by systemic inflammation. Similarly, Wells et al. suggested that age‐related changes were strongly shaped by the tissue microenvironment and not necessarily systemic inflammation [6]. Furthermore, a recent study with PBMCs from a Chinese cohort described an increase in Th1‐associated markers, particularly in males, with age (age range 20–77; [2]). This included an increase in cytotoxic CD4 T cells during aging with the highest level of inter‐individual variation in the Th1/Th2 differentiation pathway [2]. Overall, deep analysis with multi‐omics may have revealed mechanisms underlying compartmentalized immune re‐wiring. Aging induces both a Th2‐like bias in memory T cells [1], which also sits inside a larger, sex‐dependent regulatory system where Th1 and Th2 coexist and are dynamically regulated, rather than mutually exclusive endpoints [2].

## 4 Th2 Skewing During Early Aging

Among immune subsets, T cells showed the earliest and most pronounced age‐related changes, prior to older age and without inflammaging. Transcriptional and compositional changes confirmed a reduction in naïve CD8 T cells, while antigen‐specific activation and proliferation of CD4 T cells were maintained with age. CD4 memory T cells lost CXCR5 expression and exhibited reduced TCR signaling strength yet did not acquire classic senescent phenotypes in older adults as seen by others [7]. In T‐cell memory compartments, aging resulted in a Th2 bias, with increased GATA3, higher IL‐4 gene expression and IL‐4 production after TCR stimulation, while Th1/Th17 signatures were comparatively stable. These findings highlight that immune aging begins not as exhaustion but as a rewiring of early memory T cells toward this Th2 dominant state. Reduction in CXCR5 and TCR signaling strength has previously been shown to impair T follicular helper cells compromising germinal centers without altering T‐cell numbers [8]. Importantly, Th2 bias emerges prior to immunosenescence, and targeting early memory T cells could be a potential point to prevent age‐related changes in T‐cell help and tolerance. In contrast to T cells, myeloid cells showed only minor changes with age. This may be context dependent with modest and heterogeneous myeloid changes in peripheral blood [1], but more pronounced age‐related remodeling evident within tissue‐resident myeloid populations [6].

## 5 B‐Cell Responses to Vaccination Impacted During Aging

B‐cell responses displayed skewed antibody class switching and reduced vaccine responses before advanced aging, likely due to the skewed age‐related T‐cell help. Older adults showed a lower baseline hemagglutination inhibition by influenza‐specific IgG and fewer higher responders to influenza vaccinations. After vaccination, there was reduced CD27^−^ effector B‐cell frequency and skewed IGHG expression. Influenza strain‐specific IgG subclass responses were skewed with increased IgG3 post vaccination of all strains during aging, while IgG2 increased for B/Phuket, resulting in a skewed IgG2:IgG3 ratio in older adults. This is similar to previous studies describing impaired B‐cell responses to influenza vaccination [9] and B‐cell aging occurring in lymphoid niches where T–B interactions occur [6]. As IgG class switching requires T‐cell help, these findings of IgG2:IgG3 skewing, altered memory B‐cell activation, and reduced vaccine‐induced antibody function may be due to the Th2‐bias CD4 memory T cells during aging. These findings also align with literature describing age‐related B‐cell responses post vaccination of IgG2 skewing and reduced quality in responses [10].

## 6 Summary: Implications for Age‐Related Biomarkers

Together, these findings position early T‐cell transcriptional reprogramming with age as a mechanistic driver for age‐associated disease risk and reduced response to vaccination in the elderly. Th2‐skewed transcriptomes in memory T cells have also been identified in individuals at risk of autoimmune diseases, including rheumatoid arthritis and type 1 diabetes, and have been linked to neurodegenerative diseases. Others have also reported similar T‐cell transcriptional reprogramming within lymph nodes during aging, suggesting this age‐related phenotype expanded beyond just blood [6]. As this stable, nonlinear transcriptional reprogramming of T‐cell subsets occurs prior to advanced aging as shown in this study, then capturing an immune snapshot predating old age could help predict the immune landscape prior to aging, such as vaccine responses and the development of autoimmunity. The work suggests potential targets for intervention for restoring TCR signaling strength or rebalancing Th1/Th2, which could reverse age‐related changes in humoral immunity. Broadly, the use of a multi‐omic approach, immune aging metrics could help deconvolute variability in responses by older cohorts.

## Author Contributions

**Connie Jackaman:** conceptualization, writing – original draft, writing – review and editing. **Kirsten Skye Hicks:** writing – original draft, writing – review and editing, conceptualization.

## Conflicts of Interest

The authors declare no conflicts of interest.

## Data Availability

The authors have nothing to report.
